# IFIT3: a crucial mediator in innate immunity and tumor progression with therapeutic implications

**DOI:** 10.3389/fimmu.2025.1515718

**Published:** 2025-02-24

**Authors:** Rihan Wu, Hao Yang, Chunlei Liu

**Affiliations:** ^1^ Department of Radiation Oncology, Peking University Cancer Hospital (Inner Mongolia Campus) & Affiliated Cancer Hospital of Inner Mongolia Medical University, Hohhot, Inner Mongolia Autonomous Region, China; ^2^ Graduate School, Inner Mongolia Medical University, Hohhot, Inner Mongolia Autonomous Region, China; ^3^ Translational Medicine Research Center, Medical Innovation Research Division of Chinese PLA General Hospital, Beijing, China

**Keywords:** IFIT3, tumor immunity, cancer progression, innate immunity, immunotherapy

## Abstract

Interferon-Induced Protein with Tetratricopeptide Repeats 3 (IFIT3) plays a dual role in innate immunity and tumor immunity, functioning as both a viral defense molecule and a regulator of tumor progression. This review explores the mechanisms through which IFIT3 modulates immune responses, including interferon signaling, RIG-I-like receptors, and the NF-κB pathway. IFIT3 facilitates immune evasion and promotes inflammation-mediated tumor growth by regulating immune checkpoints and the tumor microenvironment, its emerging role as a target for cancer immunotherapy opens new avenues for therapeutic strategies. Finally, this paper underscores IFIT3’s potential clinical applications in the modulation of tumor immunity, highlighting the need for further research on IFIT3-targeted therapies.

## 1 Introduction

Cancer remains one of the foremost causes of death globally (1). Despite significant advancements in treatment, the complex biology of tumors and their immune escape mechanisms continue to present substantial challenges in cancer therapy. Studies have shown that tumorigenesis and progression depend on the intrinsic properties of tumor cells and are also profoundly influenced by the host immune system. In particular, there is a tight and complex network of interactions between cancer and innate immunity (2). Innate immunity is the first line of host defense against pathogen invasion (3, 4). Among interferon-inducible proteins, IFIT3 has garnered significant attention in recent years due to its dual role in innate immunity and tumor regulation (5). As a key member of the interferon-inducible protein family, IFIT3 is especially critical in the regulation of viral infections and tumor development. Recent studies have demonstrated that IFIT3 plays a crucial role in antiviral immunity, tumor microenvironment regulation, immune evasion, and inflammatory responses. Given its diverse biological functions, IFIT3 has emerged as a promising target in cancer immunotherapy (6, 7). This paper will explore the molecular mechanisms of IFIT3 across various cancers, as well as its emerging role in tumor immunity, providing a theoretical basis for future research and clinical applications.

Beyond its crucial role in cancer progression, IFIT3 is intricately involved in key immune pathways, making it a critical player in both antiviral defense and tumor-immune dynamics.

## 2 IFIT3: structure and functional roles in innate immunity

### 2.1 Structure and Function of IFIT3

IFIT3 belongs to the IFIT family of proteins in the cytoplasm and has been extensively studied for its antiviral properties. The IFIT family consists of four members, IFIT1, IFIT2, IFIT3, and IFIT5, which are clustered on human chromosome 10q23.31 (8). These proteins have no known enzymatic activity, but all of them contain unique structural patterns known as tetratricopeptide repeats (TPRs). TPRs are structural components of IFIT proteins. These TPRs consist of 3 to 16 repeated and modified tandem sequences containing 34 amino acids each. These TPRs are organized into helix-turn-helix configurations that promote their participation in protein-protein interactions leading to several protein complexes that play important roles in various biological processes in the cell (6). All four IFIT proteins have conserved structures in the N-terminal region containing the first three TPR structural domains. However, sequence conservation among IFIT proteins progressively declines towards the C-terminus, leading to increased structural diversity (9). IFIT proteins are involved in a variety of biological processes, such as cell proliferation, migration, virus-induced translation initiation, replication, and double-stranded RNA signaling. The transcription of IFIT genes can be rapidly induced by interferon (IFN) therapy and viral infection (6). The C-terminus of IFIT3 binds to the mitochondrial antiviral signaling complex and connects to NF-κB-binding kinase, which leads to the phosphorylation of IRF3 and triggers the early production of IFN-β in response to intracellular RNA viruses (9).

### 2.2 Role of IFIT3 in innate immunity

IFIT3, an integral member of the IFIT family, occupies a pivotal position in the innate immune system. It is integrated into multiple key signaling pathways, including the JAK-STAT, IFN (interferon), and Toll-like receptor (TLR)-mediated recognition pathways, the IFN (interferon) signaling pathway, and the Toll-like receptor (TLR)-mediated recognition pathway, and thus deeply participates in and strengthens the host defense mechanism. Through these pathways, IFIT3 enhances immune sensitivity and facilitates precise pathogen recognition. It participates in host defense mechanisms and plays an important role in pathogen recognition and clearance (5).

**Figure 1:**
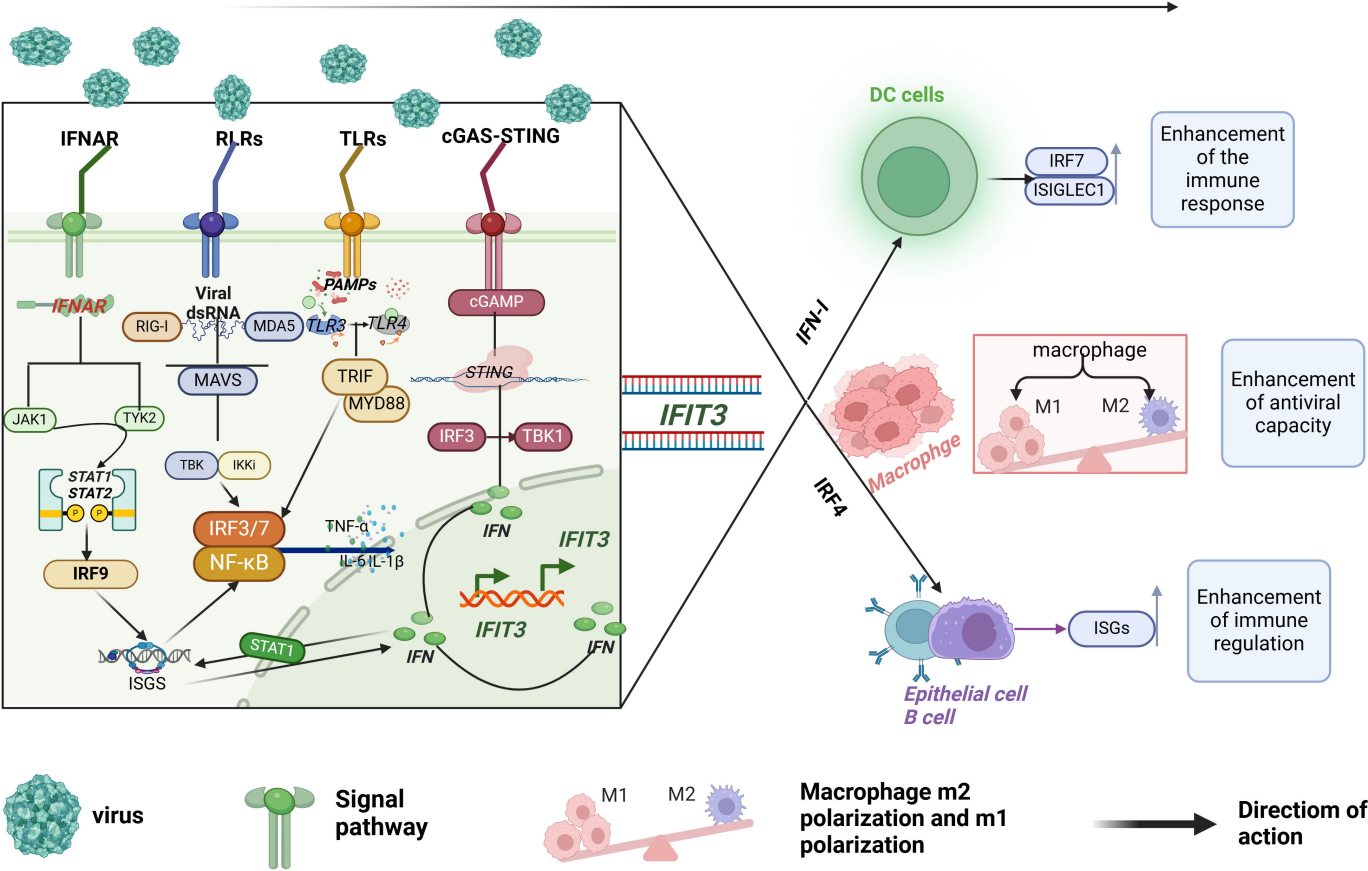
The regulatory mechanism of IFIT3 in innate immune signaling pathways. IFIT3 plays a crucial role in key immune pathways, including IFNAR, RIG-I-like receptors (RLRs), Toll-like receptors (TLRs), and cGAS-STING. IFIT3 regulates antiviral immune responses by interacting with essential signaling molecules, enhancing antigen presentation by dendritic cells, facilitating T-cell activation, and amplifying antiviral cytokine production. Additionally, IFIT3 promotes macrophage polarization and antiviral capacity, ultimately contributing to immune system regulation.

For a visual representation of these signaling pathways and IFIT3’s role within them, see (**Figure 1**). The figure illustrates how IFIT3 interacts with different immune pathways, reinforcing its central role in immune response activation through interferon signaling, innate immune receptor pathways, and inflammatory modulation.

#### 2.2.1 IFIT3 in the type I interferon signaling pathway

The IFN signaling pathway is a critical component of the innate immune system, playing a pivotal role in protecting against viral infections and regulating immune responses (10). IFN-α/β and IFN-λ initiate the expression of downstream effector genes such as IFIT3 via the JAK-STAT signaling pathway. During viral infection, IFN-α/β binds to its receptor (IFNAR) on the cell surface, activating JAK1 and TYK2 kinases. This triggers the phosphorylation and dimerization of STAT1 and STAT2. The phosphorylated STAT1/STAT2 complex then associates with IRF9, forming the ISGF3 complex, which translocates to the nucleus to drive the expression of interferon-stimulated genes (ISGs), including IFIT3 (11–13). Recent studies suggest that IFIT3 may further modulate the ISGF3 complex by influencing STAT1 phosphorylation dynamics, reinforcing the interferon response. IFIT3 is considered an early responder to viral infections (14).

Moreover, IFIT3 functions not only as a target of interferon induction but also as a regulator of interferon signaling. It has been shown that IFIT3 enhances the sustained expression of type I interferons by interacting with transcription factors, including IRF3 and IRF7, thereby amplifying the antiviral immune response (15). Through this positive feedback loop, IFIT3 enhances the interferon response, strengthening the host’s early antiviral defense mechanisms.

#### 2.2.2 IFIT3 enhances RIG-I-like receptors signaling

The RIG-I-like receptors (RLRs)—including RIG-I and MDA5—are key elements of the innate immune system responsible for recognizing viral RNA and triggering downstream antiviral signaling pathways (16). IFIT3 enhances the responsiveness of these receptors to viral RNA by interacting directly with RLRs. For example, IFIT3 binding induces structural rearrangements in RIG-I, enhancing viral RNA recognition and expediting the activation of downstream interferon signaling cascades (17). Recent studies suggest that IFIT3 stabilizes MAVS interactions with TBK1, prolonging antiviral signaling and enhancing IFN-β production.

Upon recognizing viral double-stranded RNA (dsRNA), RLRs oligomerize and form active complexes, which subsequently interact with the mitochondrial antiviral signaling protein (MAVS). MAVS functions as a hub, activating TANK-binding kinase 1 (TBK1) and IκB kinase (IKK), which in turn initiate the nuclear translocation of IRF3/7 along with transcription factors such as NF-κB. These factors regulate the expression of type I interferons and pro-inflammatory cytokines, reinforcing the antiviral state of infected and neighboring cells. IFIT3, an ISG, plays a critical role in amplifying this response by interacting with key proteins in the antiviral immune network (18, 19), Additionally, IFIT3 has been implicated in fine-tuning NF-κB activity, potentially modulating the balance between antiviral immunity and inflammatory responses.

#### 2.2.3 IFIT3 in toll-like receptor signaling pathway

Toll-like receptors (TLRs) are essential in pathogen recognition and innate immune activation (20). They detect pathogen-associated molecular patterns (PAMPs), such as viral RNA or bacterial lipopolysaccharides (LPS), and initiate downstream signaling through adaptor proteins like MyD88 or TRIF. This, in turn, activates transcription factors such as NF-κB and IRF3, leading to the induction of inflammatory cytokines and interferons, respectively (21). IFIT3, a key effector molecule in the interferon signaling pathway, not only amplifies IFN-mediated antiviral responses but also modulates TLR signaling by interacting with key adaptor proteins such as MyD88 and TRIF.

When double-stranded RNA (dsRNA) binds to TLR3, it triggers the production of interferons, initiating a cascade that includes the phosphorylation of STAT1, amplifying the expression of ISGs, including IFIT3 (14). Notably, activation of both TLR3 and TLR4 significantly upregulates IFIT3 expression, enhancing the antiviral response (14, 22). This demonstrates IFIT3’s broad role in reinforcing the cellular defense mechanisms against viral infections.

#### 2.2.4 IFIT3 and the cGAS-STING signaling pathway

The cGAS-STING pathway plays a vital role in defending against DNA viruses and certain RNA viruses. In this pathway, cyclic GMP-AMP synthase (cGAS) detects viral DNA within the cytoplasm and catalyzes the production of cyclic GMP-AMP (cGAMP), which activates the stimulator of interferon genes (STING). STING, in turn, triggers the phosphorylation of TBK1 and IRF3, leading to the robust expression of type I interferons (23, 24). IFIT3, an important effector in the type I interferon signaling pathway, enhances antiviral immunity by curbing viral replication and transmission.

Research has shown that IFIT3 is significantly elevated in monocytes from systemic lupus erythematosus (SLE) patients, where it is positively correlated with cGAS-STING pathway activity, highlighting its role in amplifying antiviral responses (25).

#### 2.2.5 Cross-regulation of IFIT3 and NF-κB signaling pathways

NF-κB is a family of transcription factors central to regulating inflammation, immune responses, and cell survival. It controls the expression of various inflammatory cytokines (e.g., IL-1, TNF-α, IL-6), chemokines, and immunoregulatory molecules (26, 27). IFIT3 has been shown to enhance immune responses through dual mechanisms. First, it promotes the expression of cytokines mediated by NF-κB, including TNF-α, IL-6, and IL-1β (28). IFIT3 participates in the activation of the NF-κB pathway, which subsequently contributes to STAT1 activation, thereby enhancing immune responses and promoting pro-inflammatory cytokine release (29). However, evidence suggests that IFIT3 is not the sole regulator of this process. A study showed that while IFIT3 knock-down reduces cytokine secretion, IFIT3 knock-out does not completely abolish pro-inflammatory cytokine release, indicating the involvement of additional pathways in this response (30).

In addition, the critical role of IFIT3 in the NF-κB signaling pathway is also notable in its complex interaction mechanism with pattern recognition receptors (PRRs). Specifically, RIG-I-like receptors (RLRs) and Toll-like receptors (TLRs), as key upstream regulatory elements of NF-κB activation, significantly enhance the transduction efficiency of the NF-κB signaling pathway through their interaction with IFIT3 (5). A study in HIV and HCV mono-infected patients revealed that IFIT3 expression was significantly upregulated in CD8 T cells from these patients, accompanied by a simultaneous upregulation of “cytokine-cytokine receptor interactions” and “NF-kappa B signaling pathway”. This was accompanied by a simultaneous upregulation of “cytokine-cytokine receptor interactions” and “NF-kappa B signaling pathway”, a chain reaction that in turn contributed to a sustained state of immune activation (31).

### 2.3 Regulation of immune cells by IFIT3

Dendritic cells (DCs), as a class of highly specialized antigen-presenting cells (APCs), occupy an important position in the regulation of adaptive immune responses, and they play an indispensable role in both the maintenance of physiological homeostasis and the modulation of immune responses in pathological states (32, 33). In a recent study, it was shown that type I interferon (IFN-I) is essential for the immune response induced in dendritic cells, and its regulated markers (IRF7, SIGLEC1), as well as induced biomarkers (IFI27, IFIT3, etc.), were validated *in vitro* and *in vivo*, which work together to build a robust immune defense system (34). Furthermore, interferon regulatory factor 4 (IRF4), a key transcription factor with hematopoietic cell specificity, significantly influences the maturation and differentiation process of immune cells. Strikingly, IRF4 can induce the expression of a specific subset of interferon-stimulated genes (ISGs) directly in epithelial cells and B-cell lines, which includes the IFIT3 gene with antiviral activity, in the absence of dependence on the type I interferon (IFN-I) signaling pathway (35). Macrophages are immune cells that receive signals from pathogens and activate the innate immune response by reprogramming gene expression (36). Overexpression of a splicing regulator called SRSF7 (formerly known as 9G8) in macrophages has been reported to result in increased IFIT3 abundance in macrophages and enhanced resistance to VSV (vesicular stomatitis virus) viral replication (37). In addition, IFIT3 was found to be included among 36 candidate genes associated with ARDS severity and also involved in M1 polarization of macrophages in one study (38). It can be seen that IFIT3, as a key antiviral protein, exhibits antiviral efficacy through its regulatory role in immune cells. IFIT3 not only inhibits the replication and transmission of viruses; but also participates in the regulation of immune cells, thereby synergistically enhancing the overall antiviral immune response of the body.

## 3 Emerging mechanisms of IFIT3 in tumor immunity

### 3.1 IFIT3 and immune checkpoint regulation

A key mechanism by which IFIT3 modulates tumor immunity is through its interaction with immune checkpoint molecules, particularly programmed death ligand 1 (PD-L1). Strong evidence indicates that IFIT3 plays a pivotal role in regulating PD-L1 expression in cancers such as non-small cell lung cancer (NSCLC) and head and neck cancer (HNC). By upregulating PD-L1, IFIT3 facilitates tumor immune evasion by suppressing cytotoxic T-cell function, thereby impairing anti-tumor immune responses (39–41). This regulation is likely mediated through the NF-κB signaling pathway, as studies have demonstrated that NF-κB activation enhances PD-L1 expression (42). Thus, targeting IFIT3 or its downstream effectors may serve as a therapeutic strategy to restore anti-tumor immunity and enhance the efficacy of immune checkpoint inhibitors, making IFIT3 a promising target for cancer immunotherapy.

### 3.2 Regulation of the tumor microenvironment by IFIT3

The tumor microenvironment (TME), which consists of cancer cells, immune cells, stromal cells, and cytokines, plays a central role in both tumor growth and immune evasion (43). IFIT3 acts as a significant regulator of the TME, influencing immune cell infiltration and the inflammatory environment within tumors. Research has shown that IFIT3 promotes the recruitment of tumor environment cells, particularly regulatory T cells (Tregs), which dampen anti-tumor immune responses (44, 45), Moreover, IFIT3 influences the polarization of tumor-associated macrophages (TAMs), promoting an M2 phenotype, which further enhances a tumor environment TME (9). Both are associated with immunosuppression in the tumor microenvironment (46, 47). These actions help foster a tumor-supportive environment by inhibiting dendritic cell maturation and reducing antigen presentation. Thus, targeting IFIT3 within the TME may offer novel strategies to reprogram the immune landscape toward tumor clearance.

In addition to regulating immune cell infiltration and polarization, IFIT3 is also involved in mediating the inflammatory responses within the TME, which play a key role in tumor progression.

### 3.3 IFIT3 and inflammation-mediated tumor progression

Chronic inflammation is a well-recognized factor contributing to cancer development and progression during tumorigenesis (48). Notably, in pancreatic ductal adenocarcinoma (PDAC), high IFIT3 expression is closely linked to elevated inflammatory markers and poor clinical outcomes. IFIT3 specifically promotes pancreatic cancer cell metastasis by inhibiting IFIT2’s pro-apoptotic effects and upregulating vascular endothelial growth factor (VEGF) and interleukin-6 (IL-6) secretion (49, 50). VEGF enhances tumor vascularization, while IL-6 fosters an inflammatory environment conducive to tumor growth. Furthermore, IFIT3 regulates intratumoral inflammatory responses by modulating key inflammatory pathways, including NF-κB and interferon regulatory factors (IRF). In particular, IFIT3 enhances NF-κB activity, which subsequently promotes the secretion of inflammatory mediators such as IL-6, TNF-α, and COX-2, driving tumor cell proliferation, resistance to apoptosis, and angiogenesis (50, 51). In addition, IFIT3 modulates pro-inflammatory macrophages, amplifying the expression of pro-inflammatory cytokines and exacerbating inflammation, which fosters a microenvironment favorable to tumor growth (52). This dual regulatory role of IFIT3 in both inflammation and tumor progression underscores its complex functions in tumor immunomodulation.

Given IFIT3’s central role in both immune evasion and inflammation-mediated tumor progression, it emerges as an attractive candidate for targeted cancer immunotherapy approaches.

### 3.4 IFIT3 as a potential target for cancer immunotherapy

**Figure 2:**
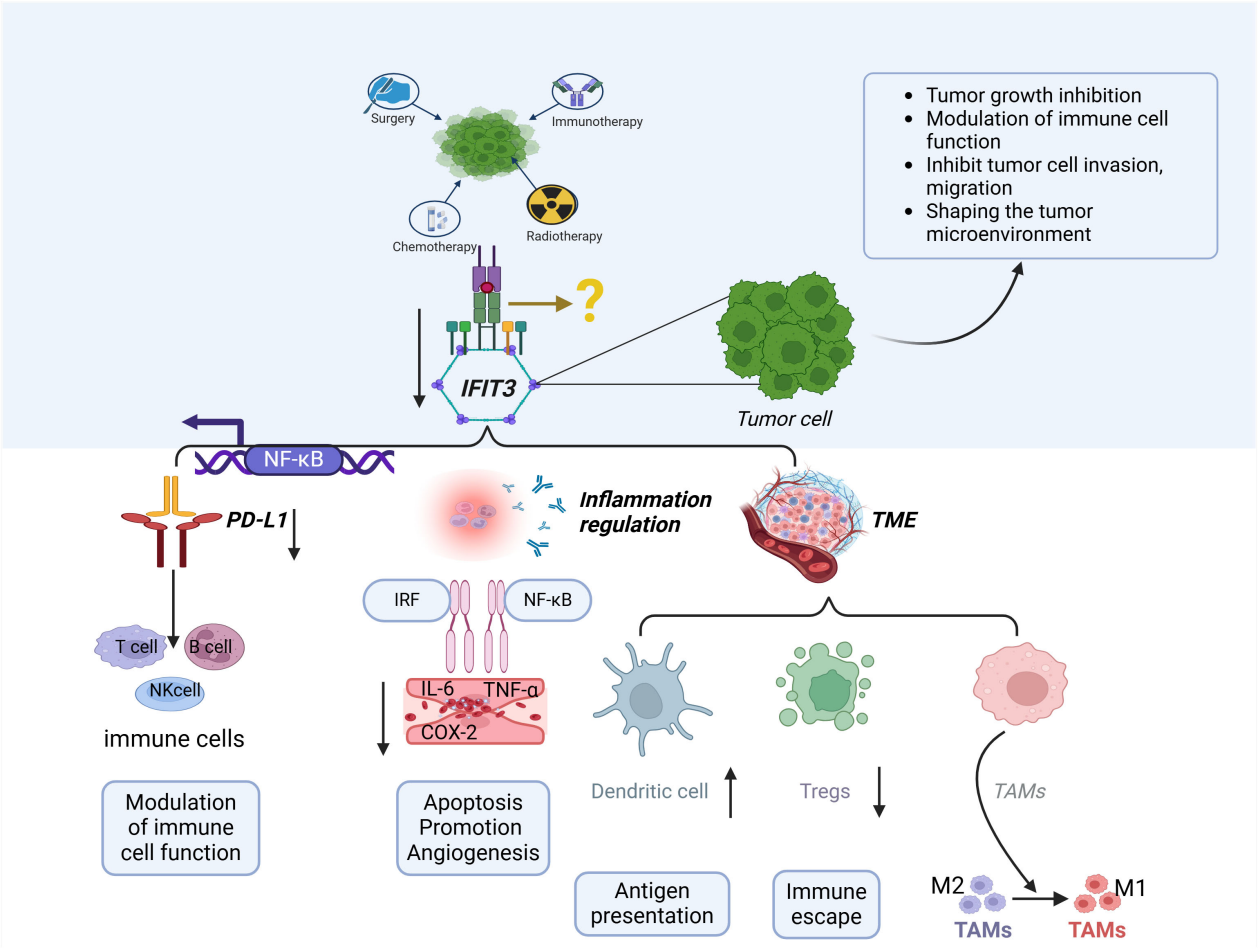
The role of IFIT3 in tumor immunity and the tumor microenvironment (TME). IFIT3 regulates tumor progression and immune evasion through multiple mechanisms. IFIT3 expression is influenced by various cancer treatments, including chemotherapy, radiotherapy, immunotherapy, and personalized therapy. Through NF-κB activation, IFIT3 upregulates PD-L1 expression, suppressing T-cell, B-cell, and NK-cell functions, thereby promoting immune evasion. In the TME, IFIT3 modulates immune cell infiltration, enhances regulatory T cell (Treg) expansion, and skews tumor-associated macrophages (TAMs) toward an M2 phenotype, fostering an immunosuppressive environment. Additionally, IFIT3 promotes inflammation via NF-κB and IRF signaling, increasing pro-inflammatory cytokines (IL-6, TNF-α, and COX-2), leading to enhanced tumor angiogenesis, apoptosis resistance, and immune escape. These findings suggest IFIT3 is a potential target for cancer immunotherapy by reshaping the immune landscape of the TME.

Studies on the involvement of IFIT3 in immune escape, tumor microenvironment regulation, and inflammation are gradually revealing its potential as a target for cancer immunotherapy (**Figure 2**). This figure illustrates how IFIT3 influences tumor progression through NF-κB activation, PD-L1 regulation, inflammatory cytokine secretion, and immune cell modulation, ultimately shaping an immunosuppressive microenvironment. Strategies aimed at inhibiting IFIT3 function or blocking its interaction with key immunoregulatory pathways, such as NF-κB and PD-L1, may enhance anti-tumor immune responses (39, 41). It has been reported in the literature that Radiotherapy, RT, is associated with a strong up-regulation of interferon-responsive genes, including IFIT3, in macrophages and dendritic cells. An in-depth study also found differences in the expression of immune checkpoints in tumors treated with RT versus those not treated with RT (53). In summary, combining IFIT3-targeted therapies with existing immunotherapies (e.g., immune checkpoint inhibitors or radiotherapy in combination with immunotherapy) may provide a synergistic approach to overcoming tumor immune resistance. Future studies should focus on validating these therapeutic strategies and evaluating their efficacy in clinical trials.

## 4 Role of IFIT3 in cancer progression: tumor-specific mechanisms

The IFIT3 gene exhibits significant expression variations across a wide range of cancer types, playing a crucial biological role. It has a profound impact on tumor initiation, progression, and patient prognosis by modulating immune response-related signaling pathways, regulating chemokine expression levels, and deeply participating in key cellular processes such as apoptosis and autophagy.

A summary of the tumor-specific mechanisms involving IFIT3 is provided in (**Table 1**), highlighting its role in different cancer types.

**Table 1:** Role of IFIT3 in cancer progression: tumor-specific mechanisms.

Cancer Type	IFIT3 Expression	Mechanism of Action	References
Oral squamous cell carcinoma (OSCC)	upregulation	Promotion of EMT) elevates p-EGFR and p-AKT levels in OSCC cells**/**LOXL2 →IFIT3→EMT/CSC→cell invasion, migration	(54–56)
Pancreatic ductal adenocarcinoma (PDAC)	upregulation	NMI promotes IFIT3 expression and tumor growth by activating the STAT3-IFIT3 signaling pathway. IFIT3 interacts with VDAC2 → enhanced chemotherapy resistance	(49, 57, 58)
Hepatocellular carcinoma (HCC)	upregulation	Silencing of IFIT3 significantly down-regulated the expression levels of IL-17 and IL-1β and inhibited the migration and invasiveness of HCC cells/Cells with low UBE2O expression and high IFIT3 expression were highly sensitive to interferon α treatment	(59, 60)
Colorectal cancer (CRC)	upregulation	ETV7 upregulation → IFIT3 upregulation → promotion of CRC cell proliferation, migration, and inhibition of apoptosis	(61, 62)
Head and neck cancer (HNC)	upregulation	IFIT3 targets PD-L1 expression through activation of the PI3K/AKT signaling pathway, which in turn regulates EMT and CSC activity	(39)
Non-small cell lung cancer (NSCLC)	Upregulation downregulation	IFIT3 overexpression enhances phosphorylation of EGFR and AKT Inhibits p53 and EMT pathway	(40, 44, 63)
prostate cancer	upregulation	Down-regulation of β6 integrin promotes IFIT3 expression in cancer cells, which in turn regulates STAT1 distribution and promotes prostate cancer progression and intercellular communication	(64)
Esophageal squamous cell carcinoma(ESCC)	upregulation	IFIT1/IFIT3+ T cells mediate immunosuppression by recruiting FoxP3+ Tregs in metastatic lymph nodes	(45, 65)
Breast cancer (BC)	upregulation	High IFIT3 expression is associated with prognosis and immune infiltration in breast cancer patients	(66–68)
bladder cancer(BLCA)	upregulation	IFIT3 showed a strong correlation with neutrophil and dendritic cell infiltration levels in the tumor microenvironment.	(69)
melanoma	upregulation	Enhancing anti-PD-1 efficacy by modulating the tumor microenvironment	(70, 71)
thyroid cancer	upregulation	Combined bioinformatic synthesis results in IFIT3 expression is associated with poor prognosis in thyroid cancer	(72)
leukemia	Upregulation downregulation	Impact on the tumor microenvironment (TME), high expression of IFIT3 is associated with poor prognosis in acute myeloid leukemia (AML)**/**IFIT1/IFIT3 inhibits Bcl-2 through pyroptosis In acute promyelocytic leukemia (APL), the PML-RAR fusion protein inhibits the RIG-G (i.e. IFIT3) expression and inhibits disease progression	(73–76)
myeloma (medicine)	Upregulation	IFIT3 indirectly inhibits MYC, targets the MYC-IRF4 axis, and activates the immune/interferon pathway	(74, 77)
ovary cancer	downregulation	Negative regulation of cell proliferation, cellular immunity	(78, 79)
Nasopharyngeal Carcinoma NPC	Upregulation	IFIT3 plays a role in the early diagnosis of nasopharyngeal cancer	(80)

### 4.1 Oral squamous cell carcinoma

Oral squamous cell carcinoma (OSCC) accounts for more than 90% of all oral cancer cases, with a five-year survival rate of less than 50%, ranking 16th in global cancer mortality (81, 82). Notably, a previous study has reported that the expression levels of IFIT3 are significantly upregulated in OSCC (54). IFIT3 overexpression has been identified as a major contributor to epithelial-mesenchymal transition (EMT) in OSCC, where it promotes tumor invasiveness. Inhibition of this pathway has shown potential therapeutic benefits, making IFIT3 a viable target for future drug development (55). A significant positive correlation between LOXL2 expression and the overexpression of IFIT1 and IFIT3 was observed in human OSCC tissues. This suggests that LOXL2 may regulate the expression of IFIT3, which has important implications for tumor progression. Later in the paragraph, LOXL2’s role in modulating the tumor microenvironment is discussed in more detail. Additional studies revealed that Lysyl oxidase-like 2 (LOXL2) expression in OSCC tissues was significantly correlated with tumor clinical stage, and lymph node metastasis patient overall survival. Human OSCC TW2.6 (TW2.6/LOXL2) cells overexpressing LOXL2 exhibit enhanced migration, invasion, epithelial-mesenchymal transition (EMT), and cancer stem cell (CSC) phenotypes. Notably, in LOXL2-overexpressing cells, LOXL2 increased the levels of interferon-inducible proteins IFIT1 and IFIT3, which are key downstream components involved in the migration, invasion, EMT, and CSC phenotypes of TW2.6 cells (56).

IFIT3 overexpression has been identified as a major contributor to epithelial-mesenchymal transition (EMT) in OSCC, where it promotes tumor invasiveness. Inhibition of this pathway has shown potential therapeutic benefits, making IFIT3 a viable target for future drug development. In summary, IFIT3 overexpression plays a crucial role in OSCC progression.

### 4.2 Pancreatic ductal adenocarcinoma

Pancreatic ductal adenocarcinoma (PDAC) is one of the most lethal malignant tumors worldwide (83). with recent research revealing the significantly high expression of IFIT3 in aggressive PDAC cells. This abnormally high expression not only strengthens the anti-apoptotic ability of PDAC cells but also significantly increases their resistance to chemotherapeutic drugs, directly correlating with shorter patient survival (57). IFIT3 plays a key role in the regulation of mitochondria-mediated apoptosis during chemotherapy. Knockdown of IFIT3 expression effectively weakened PDAC cells’ resistance to a range of chemotherapeutic agents, including gemcitabine, paclitaxel, and FOLFIRINOX, whereas overexpression of IFIT3 significantly promoted the development of resistance. Immunoprecipitation studies revealed a direct interaction between IFIT3 and mitochondrial voltage-dependent anion channel protein 2 (VDAC2), a key regulator of the mitochondria-associated apoptosis pathway. IFIT3 forms a protective barrier against chemotherapy-induced apoptotic signals in PDAC cells by stabilizing the binding of VDAC2 to O-GlcNAc transferase (49). Additionally, studies showed that N-myc and STAT interactor (NMI) promoted IFIT3 expression and accelerated tumor growth and migration by activating the STAT3-IFIT3 signaling pathway. NMI-mediated upregulation of IFIT3 plays a central role in PDAC cell resistance to chemotherapeutic drugs such as gemcitabine, providing a potential target for novel therapeutic strategies (58).

### 4.3 Hepatocellular carcinoma

Hepatocellular carcinoma (HCC) is one of the most prevalent malignant tumors, accounting for approximately 90% of liver cancer cases and representing a leading cause of cancer-related deaths globally (84, 85). Research into the molecular mechanisms underlying HCC has shown that the expression of CXCL11 in cancer-associated fibroblasts (CAFs) is significantly upregulated compared to other molecules, a trend that is also evident in both cirrhotic and HCC tissues when compared to normal liver tissues. Moreover, analysis of non-metastatic and metastatic HCC tissue samples has revealed markedly elevated mRNA levels of IFIT1 and IFIT3 in comparison to paraneoplastic tissues (59).

CircUBAP2, a circular RNA, is highly upregulated in the majority of HCC tissues and is associated with poor patient prognosis. HCC patients exhibiting high levels of circUBAP2 expression tend to have greater vascular invasion and worse differentiation (86). CircUBAP2 has been identified as a key regulatory molecule that modulates the expression of IFIT1 and IFIT3. Acting as a competitive endogenous RNA (ceRNA), it sponges miR-4756, thereby preventing the inhibition of IFIT1 and IFIT3 expression. This regulatory axis is critical in HCC, as circUBAP2 influences both immune responses and tumor progression (59).

Additionally, studies have demonstrated that silencing IFIT1 and IFIT3 significantly downregulates IL-17 and IL-1β expression, impairing the migration and invasiveness of HCC cells (59). Notably, the ubiquitin-binding enzyme UBE2O, which is strongly correlated with HCC prognosis, plays an essential role in the ubiquitination of IFIT3. Findings indicate that HCC cells with high levels of UBE2O expression and low levels of IFIT3 expression exhibit strong resistance to interferon α treatment, whereas those with low UBE2O expression and high IFIT3 expression display heightened sensitivity to interferon α (60).

### 4.4 Colorectal cancer

Colorectal cancer (CRC), the third most prevalent type of cancer globally, accounts for approximately 1.9 million new cases annually, constituting one-tenth of all new cancer diagnoses (87). Recent studies have uncovered the pivotal role of ETS variant transcription factor 7 (ETV7) in CRC pathogenesis. ETV7 expression is significantly upregulated in CRC tissues and cells, promoting abnormal proliferation, migration, and cell cycle acceleration while inhibiting the normal apoptotic processes of CRC cells. Notably, ETV7 expression levels in CRC patients were positively correlated with IFIT3, with ETV7 enhancing IFIT3 transcriptional activity, mRNA levels, and protein expression in CRC cells (61).

### 4.5 Head and neck cancer

Head and neck squamous cell carcinoma (HNSCC), the most prevalent malignant tumor of the upper respiratory and digestive tracts, accounts for the vast majority of head and neck cancer (HNC) cases and ranks as the seventh most common cancer worldwide. It is characterized by high aggressiveness, significant metastasis propensity, and a high recurrence rate (88). Recent bioinformatics analyses, cellular experiments, and animal models have revealed the critical role of IFIT3 in HNSCC. IFIT3 is highly expressed in HNSCC tissues, and its abnormal overexpression is directly correlated with poor prognosis in patients with clinical stage IV or pathological grade 3, as reflected by significantly reduced survival rates. Additional studies have investigated the mechanisms through which IFIT3 promotes malignant progression in HNSCC. IFIT3 specifically targets programmed death ligand 1 (PD-L1) expression by activating the PI3K/AKT signaling pathway, thereby regulating the epithelial-mesenchymal transition (EMT) and cancer stem cell (CSC) activity. These molecular cascade reactions represent a key mechanism through which IFIT3 drives tumor progression and metastasis in HNSCC (39).

### 4.6 Non-small cell lung cancer

Lung cancer has one of the highest incidence and mortality rates globally. In 2023, the American Cancer Society estimated more than 1.8 million new cases and 1.6 million deaths worldwide (89) Non-small cell lung cancer (NSCLC) constitutes the majority of lung cancer cases (85% of patients) (90).

The epidermal growth factor receptor (EGFR) signaling pathway plays a crucial role in lung cancer development, with EGFR mutations and overexpression being key features of NSCLC. This signaling influences several biological processes, including cell proliferation, differentiation, and survival (91). Some studies have shown that the knockdown of IFIT1 or IFIT3 inhibits NSCLC cell proliferation and invasion and promotes apoptosis, suggesting that IFIT3 acts as an oncogene in NSCLC progression. Furthermore, IFIT3 overexpression significantly enhances the phosphorylation of EGFR and AKT, regulating multiple effector molecules in the EGFR pathway, and thus plays a multilevel role in cell proliferation and survival (40). A recent study on lung adenocarcinoma (LUAD) identified cyclic RNA Circ_BBS9 as a tumor suppressor. Overexpression of circ_BBS9 inhibited LUAD cell proliferation and promoted ferroptosis. IFIT3, which directly interacts with circ_BBS9, is involved in immune infiltration and the formation of the immune microenvironment. It may serve as a diagnostic biomarker by regulating ferroptosis and the immune microenvironment through competitive binding to miR-7150 (44).

Interestingly, a study found that Rig-G (an alias of IFIT3) expression was often downregulated in lung cancer, and its low levels were strongly associated with poor prognosis (63). Further exploration revealed that Rig-G overexpression effectively inhibited tumor growth and migration in lung cancer cells and animal models, highlighting its potential as a tumor suppressor capable of significantly slowing lung cancer progression and metastasis. In A549 lung cancer cells, Rig-G overexpression significantly suppressed p53 downstream genes. Upregulation of Rig-G promoted the expression of E-cadherin and p21, enhancing cell adhesion and growth inhibition; while suppressing the expression of vimentin, a key EMT marker. Interestingly, however, the intervention of the p53 inhibitor Pifithrin-α (PFTα) significantly attenuated the inhibitory effect of Rig-G on p53 and EMT pathways in lung cancer cells. Overall, Rig-G exerts its tumor suppressor effects in lung cancer through p53-dependent pathways (63).

### 4.7 Prostate cancer

Prostate cancer (PrCa) is one of the leading causes of cancer morbidity and mortality in men worldwide (92). It is estimated that 288,300 new cases of prostate cancer (PrCa) will be diagnosed in the U.S. in 2023, accounting for 29% of new cancer cases in men. It is the most common cancer among men in the United States, and the current lifetime risk of prostate cancer in men is 1 in 8 (93). In prostate cancer (PrCa) study, downregulation of the β6 integrin subunit was found to significantly promote IFIT3 expression in PrCa cells and their released small extracellular vesicles (sEVs), suggesting that IFIT3 may be negatively regulated by β6 integrin. Meanwhile, there was a complex interaction between IFIT3 and STAT1, and although both were highly expressed in PrCa cells, IFIT3 was the only factor secreted into sEVs. Further, the reduction of IFIT3 resulted in STAT1 enrichment in sEVs and decreased intracellular STAT1 levels, revealing a critical role of IFIT3 in regulating STAT1 distribution. These findings emphasize the importance of IFIT3 in PrCa progression and intercellular communication (64).

### 4.8 Other cancers

With ongoing research, the role of IFIT3 in cancer has become increasingly prominent. In addition to the known fields, the critical role of IFIT3 in a variety of cancer types, including but not limited to esophageal squamous cell carcinoma (45, 65), myeloma, leukemia (73, 74), breast cancer (66), nasopharyngeal carcinoma (80), bladder carcinoma (69), thyroid carcinoma (72), and melanoma (70), has been further revealed utilizing comprehensive bioinformatic analyses and other means. These studies have shown that IFIT3 can significantly regulate the proliferation rate, migration and invasion potential, and the dynamic balance of immune responses of tumor cells, as well as affect the sensitivity of cancer cells to chemotherapeutic agents, and thus it is considered as an indispensable key regulator in the mechanism of the occurrence and development of these cancers.

## 5 Conclusion

As a key immunoregulatory molecule, IFIT3 has demonstrated its important role in tumorigenesis, development, and immune escape, and has become a hotspot of tumor immunity research in recent years. This review provides a comprehensive overview of IFIT3’s multifaceted roles in cancer progression, immune evasion, and drug resistance. By regulating key processes such as cell proliferation, invasion, immune checkpoint modulation, and inflammation, IFIT3 has emerged as a critical player in tumor immunity. Its involvement in pathways such as interferon signaling, RIG-I-like receptors, Toll-like receptors, cGAS-STING, and NF-κB highlights its potential as a target for cancer immunotherapy. Future research should focus on uncovering IFIT3’s interactions with additional immune checkpoints across various cancer types and investigating the efficacy of IFIT3-targeted therapies in clinical settings.

Of interest is the unique role of IFIT3 in the remodeling of the tumor microenvironment, the regulation of inflammatory response, and the modulation of immune checkpoint molecules, suggesting that it has an important potential for clinical application in tumor immunotherapy. Future studies should explore more deeply the regulatory role of IFIT3 in other immune checkpoint molecules, especially the differential expression in different cancer types. Meanwhile, it will be clinically important to evaluate the effect of IFIT3 inhibitors or targeted modulation strategies against tumors in conjunction with the latest advances in immunotherapy. Integrating IFIT3-targeted therapies with established immunotherapies, such as PD-1/PD-L1 inhibitors, holds promise for novel cancer treatment strategies. In addition, the cross-regulation of IFIT3 with signaling pathways such as NF-κB and cGAS-STING should be the focus of future studies to understand its multiple roles in tumors fully.
